# Bacterial superglue enables easy development of efficient virus-like particle based vaccines

**DOI:** 10.1186/s12951-016-0181-1

**Published:** 2016-04-27

**Authors:** Susan Thrane, Christoph M. Janitzek, Sungwa Matondo, Mafalda Resende, Tobias Gustavsson, Willem Adriaan de Jongh, Stine Clemmensen, Will Roeffen, Marga van de Vegte‑Bolmer, Geert Jan van Gemert, Robert Sauerwein, John T. Schiller, Morten A. Nielsen, Thor G. Theander, Ali Salanti, Adam F. Sander

**Affiliations:** Centre for Medical Parasitology at the Department of Immunology and Microbiology, University of Copenhagen, Copenhagen, Denmark; Department of Infectious Diseases, Copenhagen University Hospital, Copenhagen, Denmark; Kilimanjaro Clinical Research Institute, KCMC, Moshi, Tanzania; ExpreS2ion Biotechnologies, SCION-DTU Science Park, Hørsholm, Denmark; Department of Medical Microbiology, Radboud University Medical Center, Nijmegen, The Netherlands; Laboratory of Cellular Oncology, National Cancer Institute, National Institutes of Health, Bethesda, MD USA

## Abstract

**Background:**

Virus-like particles (VLPs) represent a significant advance in the development of subunit vaccines, combining high safety and efficacy. Their particulate nature and dense repetitive subunit organization makes them ideal scaffolds for display of vaccine antigens. Traditional approaches for VLP-based antigen display require labor-intensive trial-and-error optimization, and often fail to generate dense antigen display. Here we utilize the split-intein (SpyTag/SpyCatcher) conjugation system to generate stable isopeptide bound antigen-VLP complexes by simply mixing of the antigen and VLP components.

**Results:**

Genetic fusion of SpyTag or SpyCatcher to the N-terminus and/or C-terminus of the *Acinetobacter* phage AP205 capsid protein resulted in formation of stable, nonaggregated VLPs expressing one SpyCatcher, one SpyTag or two SpyTags per capsid protein. Mixing of spy-VLPs with eleven different vaccine antigens fused to SpyCatcher or SpyTag resulted in formation of antigen-VLP complexes with coupling efficiencies (% occupancy of total VLP binding sites) ranging from 22–88 %. In mice, spy-VLP vaccines presenting the malaria proteins Pfs25 or VAR2CSA markedly increased antibody titer, affinity, longevity and functional efficacy compared to corresponding vaccines employing monomeric proteins. The spy-VLP vaccines also effectively broke B cell self-tolerance and induced potent and durable antibody responses upon vaccination with cancer or allergy-associated self-antigens (PD-L1, CTLA-4 and IL-5).

**Conclusions:**

The spy-VLP system constitutes a versatile and rapid method to develop highly immunogenic VLP-based vaccines. Our data provide proof-of-concept for the technology’s ability to present complex vaccine antigens to the immune system and elicit robust functional antibody responses as well as to efficiently break B cell self-tolerance. The spy-VLP-system may serve as a generic tool for the cost-effective development of effective VLP-vaccines against both infectious- and non-communicable diseases and could facilitate rapid and unbiased screening of vaccine candidate antigens.

**Electronic supplementary material:**

The online version of this article (doi:10.1186/s12951-016-0181-1) contains supplementary material, which is available to authorized users.

## Background

Active vaccination against infectious diseases has been one of the most effective medical interventions in human history with a tremendous impact on global health. Due to safety-, manufacturing- and reproducibility concerns, global vaccine development has gradually turned its focus away from whole-pathogen based vaccines and towards recombinant subunit vaccines based on defined antigen components [1]. The effectiveness of simple subunit vaccines is, however, considerably inferior to that of whole-pathogen-based vaccines and the successful development of soluble proteins as vaccine candidates has in many cases been a disappointment. The low immunogenicity of soluble protein antigens has been attributed to their small size (<10 nm), susceptibility to proteolytic degradation, and a low capacity for activating the innate immune system. Virus-like particles (VLPs) represent a specific class of particulate subunit vaccines, which are highly immunogenic due to sharing key characteristics with live viruses [2]. Several VLP-vaccines have already been commercialized *e.g.* Engerix (hepatitis B virus) and Cervarix (human papillomavirus) by GlaxoSmithKline, Recombivax HB (hepatitis B virus) and Gardasil (human papillomavirus) by Merck, and Hecolin (hepatitis E virus) by Xiamen Innovax [3]. VLPs are safe non-replicating shells consisting solely of viral structural proteins that, when overexpressed, self-assemble into dense multi-protein arrays with icosahedral or rod-like structures. The size of VLPs (20–200 nm) allows for direct drainage into lymph nodes and is optimal for uptake by antigen-presenting cells and cross-presentation [4]. Their highly repetitive surface structures moreover enable complement fixation and B cell receptor clustering, altogether leading to the activation of the innate immune system, greater B cell activation and ultimately increased antibody production [4–6]. Importantly, it has been established that hetorologous antigens displayed on VLPs can assume a similar immunogenicity as the underlying particle, creating a strong rational for using VLPs as antigen-presenting platforms to increase immune responses against otherwise poorly immunogenic antigens [2, 7]. Antigen display has traditionally been achieved by either genetic fusion of heterologous epitopes into the self-assembling coat protein or by conjugation to preassembled VLPs. Genetic fusion of smaller peptides (often single epitopes) has in several cases been successful, whereas insertion of larger sequences generally prevents VLP-assembly [2, 8, 9]. Even if VLP-assembly is achieved, chimeric particles are often instable and the functional conformation of the inserted epitope may not be retained. Consequently, the genetic fusion approach is inevitably based on substantial trial-and-error optimization and is largely restricted to continuous epitopes thus requiring the pre-identification of such determinants in the target-antigen. Chemical cross-linking chemistry has been employed to conjugate target antigens to pre-assembled VLPs by stimulating covalent linkage between reactive amino acid side chains in the antigen and coat protein sequences, respectively [10, 11]. Complex antigens, however, generally present multiple reactive sites hampering consistent directional coupling of the antigen to the VLP required for optimal epitope display. In addition, such chemical reactions often result in a lower than optimal density of the VLP-displayed antigen [10, 12]. Other strategies, involving non-covalent antigen-VLP conjugation have also been pursued, each with individual advantages and disadvantages [13, 14]. The most successful general approach was developed by Cytos Biotech and involves the terminal addition of a reactive Cysteine residue to the target-antigen followed by addition of a hetero-bifunctional cross-linker to mediate coupling between the reactive sulfhydryl group (Cys) and the N-term of Lysine residues exposed on the surface of Qbeta VLPs [10, 15]. Several promising VLP-vaccines have been developed by this technology, although this method suffers from an inconsistent ability to display complex antigens with conformation-dependent epitopes. Therefore, there is a strong interest in developing new methods to obtain optimal VLP-display for complex target-antigens. Herein we report the use of the split-intein (SpyTag/SpyCatcher) conjugation system [16] to facilitate conjugation of target antigens to VLPs under physiological conditions. This conjugation system takes advantage of the spontaneous formation of an isopeptide bond between a Lys and an Asp present in two split units of the *Streptococcus pyogenes* fibronectin-binding protein FbaB. These split units consist of a peptide (SpyTag) and a protein (SpyCatcher), which in solution interact to form a highly stable amide bond. The irreversible reaction occurs within minutes and so this technology offers a simple way to conjugate antigen to VLPs. We developed a panel of genetically modified *Acinetobacter* phage AP205 VLPs displaying either the SpyCatcher protein (116 amino acids) or the SpyTag peptide (13 amino acids) in regular arrays. We also engineered an AP205 VLP presenting two SpyTags per VLP subunit (2xSpyTag-VLP). We characterized these spy-VLPs in terms of stability and antigen display capacity using a variety of antigens and discuss how the different spy-VLPs can be used to increase the versatility of the spy-VLP display system. Using two malaria antigens, we demonstrate that the spy-VLP system elicited high levels of high affinity IgG, which effectively inhibited key processes in the parasite development. Finally, we show that B cell self-tolerance can be overcome by the spy-VLP system, which effectively induced IgG against a range of self-antigens including PD-L1, CTLA-4 and IL-5. Thus, the data demonstrate the broad usability of the spy-VLP platform and validate its ability to facilitate strong functional antibody responses against complex vaccine antigens.

## Results

### Development, expression and characterization of spy-VLPs

**Fig. 1:**
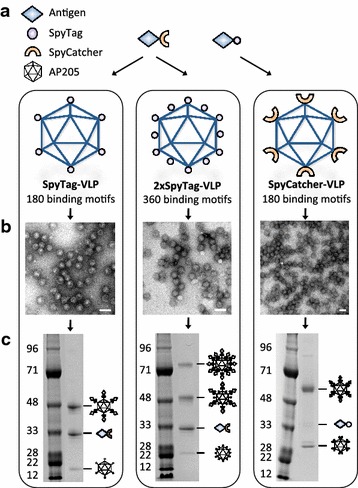
The spy-VLP antigen display platform. **a** Three types of spy expressing VLPs were constructed by genetic fusion of SpyTag or SpyCatcher to the virus-like particle (VLP)-forming AP205 capsid protein. (1) “SpyTag-VLP” had the SpyTag fused to the N-terminus of the AP205 capsid protein and present 180 potential SpyCatcher-antigen binding motifs (2) “2xSpyTag-VLP” had SpyTag fused to both the N- and C-terminus of the AP205 capsid protein and present 360 potential SpyCatcher-antigen binding motifs; (3) “SpyCatcher-VLP” had SpyCatcher fused to the N-terminus of the AP205 capsid protein and present 180 potential Spytag-antigen binding motifs. **b** Transmission electron microscopy (TEM) images showing the SpyTag-VLP, 2xSpyTag-VLP and SpyCatcher-VLP. Purified spy-VLP samples were placed on carbon, adsorbed to a grid and negatively stained with 2 % phosphotungstic acid. *Scale bar* 50 nm. Images show uniform, non-aggregated particles of approximately 30 nm (SpyTag-VLP and 2xSpyTag-VLP) and 42 nm (SpyCatcher-VLP). **c** Reduced SDS-PAGE gels loaded with VLP vaccines demonstrating that vaccine proteins had formed covalent bonds to the AP205 capsid protein. *Left panel* shows that mixing of SpyTag-VLPs with SpyCatcher-IL-5 resulted in three protein bands corresponding to the size of an antigen-VLP capsid protein conjugate (48 kDa) (*top*), uncoupled vaccine antigen (33 kDa) (*middle*) and unconjugated SpyTag-VLP capsid protein (16.5 kDa) (*bottom*). The *middle panel* shows that mixing of 2xSpyTag-VLP with SpyCatcher-IL-5 resulted in four protein bands representing; a conjugate of two vaccine antigens bound to each end of a 2xSpyTag-VLP capsid protein (83 kDa), a conjugate of the 2xSpyTag-VLP capsid protein and a single vaccine antigen (48 kDa), uncoupled vaccine antigen (33 kDa) and unconjugated 2xSpyTag-VLP capsid protein (18.5 kDa). The *left panel* shows that mixing of SpyCatcher-VLP with PD-L1-SpyTag resulted in three protein bands representing; an antigen-VLP capsid protein conjugate (50 kDa), uncoupled vaccine antigen (33 kDa) and unconjugated SpyCatcher-VLP capsid protein (27 kDa)

A panel of SpyTag or SpyCatcher presenting VLPs was designed based on the *Acinetobacter* phage AP205 coat protein. Expression of this protein in *Escherichia coli* results in the assembly of 29 nm icosahedral (T = 3) VLPs consisting of 180 subunits [17]. Precisely, the 116 amino acid SpyCatcher sequence was fused to the N-terminus (SpyCatcher-VLP) of the AP205 coat protein (Gene ID: 956335). In addition, the 13 amino acid SpyTag peptide was fused to N-terminus (SpyTag-VLP) or to both N- and C-terminus (2xSpyTag-VLP) of the AP205 coat protein (Fig. 1a). Recombinant *E. coli* expression of spy-AP205 coat proteins was confirmed by SDS-PAGE analysis of fractions collected following density gradient ultracentrifugation. Reduced SDS-PAGE showed pure protein bands of expected sizes (Additional file 1: Figure S1). VLP-assembly of each spy-AP205 coat proteins was evaluated by transmission electron microscopy (TEM) (Fig. 1b) and dynamic light-scattering (DLS) analysis. For all recombinant particles the DLS analysis revealed a homogenous population of non-aggregated particles with an average estimated size of 36 nm [Pd = 12.1] (SpyTag-VLP), 42 nm [Pd = 21.6] (2xSpyTag-VLP) and 43 nm [Pd = 9.7] (SpyCatcher-VLP). In comparison, unmodified AP205 VLPs were determined by DLS to have an average size of 35 nm [Pd = 10.7].

To test if the recombinant spy-VLPs could form a covalent interaction with an antigen through their SpyTag or SpyCatcher, individual spy-VLPs were mixed with antigen fused to the corresponding binding-partner and formation of antigen-VLP subunit conjugates was subsequently confirmed by SDS-PAGE analysis. For all spy-VLP types and all tested antigens the SDS-PAGE revealed the occurrence of a protein band matching the combined size of the antigen and VLP subunit, as exemplified in Fig. 1c. Mixing of SpyTag- or SpyCatcher-fused antigen with unmodified AP205 VLPs did not produce this banding pattern (data not shown).

### Versatility of the spy-VLP platform

To explore the versatility of the spy-VLP antigen display system we cloned and expressed 11 vaccine candidate antigens genetically fused to either a SpyTag or a SpyCatcher (Additional file 2: Table S1). The panel of spy-antigens, representing very diverse proteins with respect to origin, structure and size (14.5–118 kDa) included; (a) malaria proteins: CSP, CIDR, VAR2CSA, and Pfs25, which are expressed at different developmental stages of the complex life cycle of *Plasmodium falciparum* and used in different vaccine strategies to reduce malaria transmission or disease [18, 19]; (b) the *Mycobacterium tuberculosis* protein, Ag85A, in development for a tuberculosis vaccine; (c) mouse proteins involved in cancer (CTLA-4, PD-L1, Survivin and HER2), asthma/allergy (IL-5) or cardiovascular disease (PCSK9). The latter self-proteins are targets of therapies employing monoclonal antibody. The vaccine antigens were mixed with corresponding spy-VLPs and the antigen coupling efficiency (% occupancy of total VLP binding sites) and antigen display capacity (number of antigens per VLP) was estimated for each reaction by SDS-PAGE densitometric analysis. Standard molar mixing ratio was 1:1.5 (VLP:antigen) and reactions occurred over night at 4 °C. A similar coupling efficiency was observed using three-hour incubations at 37 °C (data not shown). Overall, antigen-coupling efficiencies ranged from 22–88 %, corresponding to 40–159 antigens displayed per VLP (Table 1).

**Table 1:** Estimation of antigen coupling efficiency

Spy-VLP	Potential binding motifs	Spy-antigen	Antigen size (kDa)	Coupling efficiency (%)	Display capacity (antigens/VLP)
SpyTag	180	SpyCatcher-CIDR	32	76	*136*
SpyTag	180	SpyCatcher-IL-5	33	77	*138*
SpyTag	180	SpyCatcher-Ag85A	48	75	*134*
SpyTag	180	CSP-SpyCatcher	53	48	*86*
SpyTag	180	SpyCatcher-HER2	83	22	*40*
SpyTag	180	PCSK9-SpyCatcher	84	23	*42*
2xSpyTag	360	SpyCatcher-Survivin	30	52 (104)^a^	*187*
2xSpyTag	360	SpyCatcher-IL-5	33	54 (107)^a^	*193*
2xSpyTag	360	Pfs25-SpyCatcher	40	30 (61)^a^	*109*
SpyCatcher	180	CTLA-4-SpyTag	15	88	*159*
SpyCatcher	180	PD-L1-SpyTag	27	45	*81*
SpyCatcher	180	SpyTag-VAR2CSA	118	34	*61*

^a^% occupancy of total VLP subunits: number of displayed antigens/total number of VLP subunits (=180) × 100

There was a negative correlation between antigen size and the number of antigens bound per VLP (Spearman Rank Order Correlation Coeff. = −0.75, P = 0.02) (the 2xSpyTag-VLP constructs were excluded from the analysis). There was no significant difference in the antigen coupling efficiency in SpyTag-VLP and SpyCatcher-VLP reactions. However, the estimated coupling capacity was higher for the reaction between SpyCatcher-IL-5 and 2xSpyTag-VLP (193 antigens per VLP) compared with mixing similar amounts of SpyCatcher-IL-5 with SpyTag-VLP (138 antigens per VLP) (Table 1; Additional file 3: Figure S2D).

### Immunogenicity of spy-VLP vaccines

To assess the immunogenicity of spy-VLP delivered antigens, we tested two clinically relevant malaria proteins, Pfs25 and VAR2CSA, and evaluated humoral responses in mice after intramuscular immunizations. Pfs25 is expressed on the *P. falciparum* ookinete surface within the mosquito. Immunization with recombinant Pfs25 formulated in Montanide ISA51 induced Pfs25-specific antibodies with capacity to block parasite infectivity to mosquitoes in a Phase 1 human clinical trial but the vaccine had unacceptable side effects [20]. The Pfs25 antigen is poorly immunogenic by itself and development of an effective transmission-blocking Pfs25 vaccine has been hampered by the requirement of a strong adjuvant [20–22]. SpyCatcher was fused to the C-terminus of Pfs25 and Pfs25-SpyCatcher was expressed and purified from *E. coli* SHuffle^®^ cells. This enabled high level expression of soluble correctly folded Pfs25-SpyCatcher as verified by binding of the transmission-blocking anti-Pfs25 monoclonal antibody, mAb 4B7 [23] (Additional file 4: Figure S3).

VAR2CSA is a unique member of the *P. falciparum* erythrocyte membrane protein 1 (PfEMP1) protein family. This protein binds parasite-infected erythrocytes to placental chrondroitin sulphate A (CSA) [24]. Anti-VAR2CSA antibodies can prevent this binding [25, 26], and clinical testing of a protein-based VAR2CSA vaccine to protect women against placental malaria has been initiated [27]. SpyTag was genetically fused to the N-terminus of the CSA binding domain of VAR2CSA (domains DBL1-ID2a) and expressed and purified from *E. coli* SHuffle^®^ cells. The protein expressed well and was folded correctly as measured by its binding to decorin, as described in [28].

#### Spy-VLP vaccine induced IgG titers

**Fig. 2:**
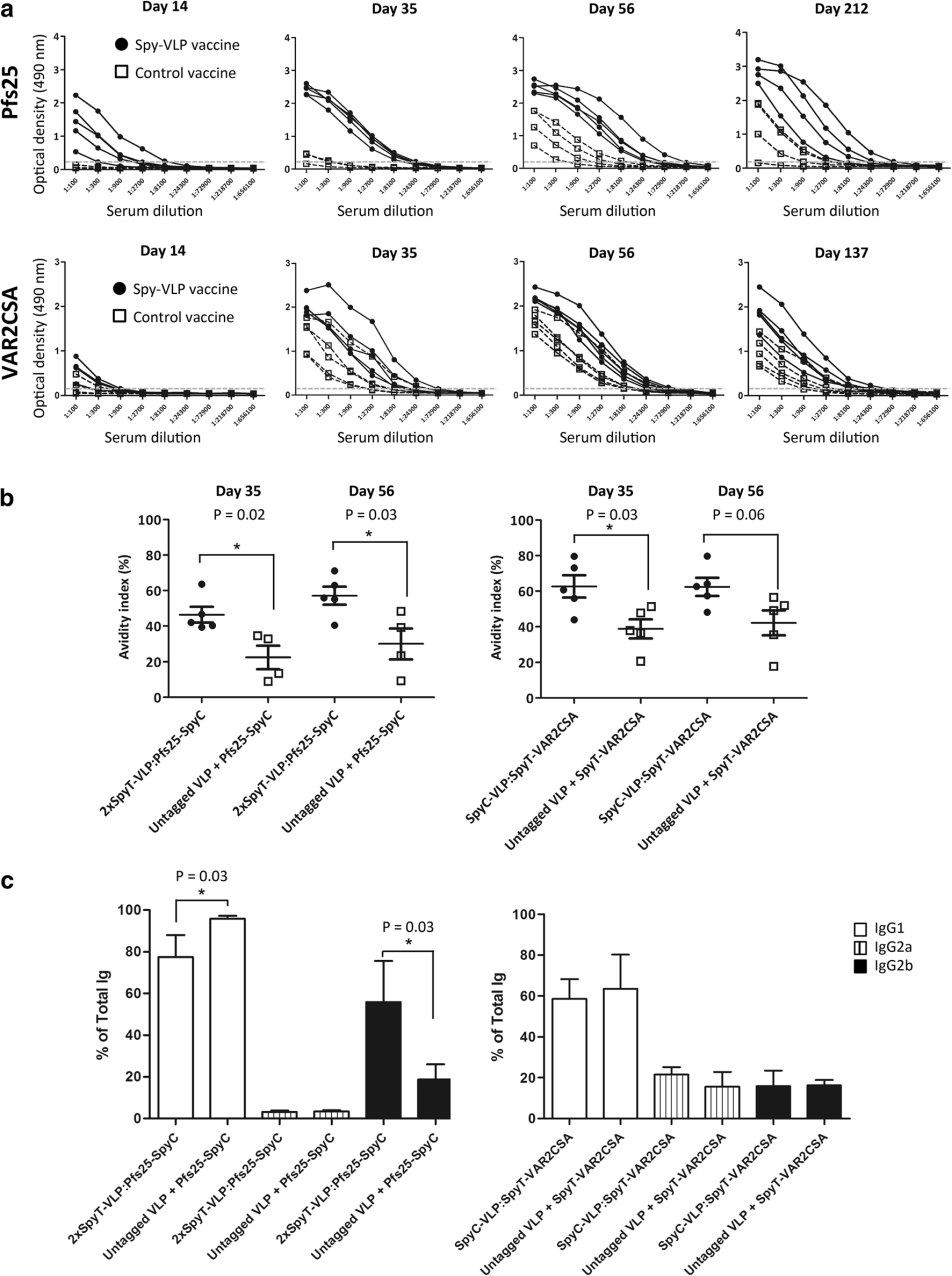
Antigen-specific IgG levels in mice after immunization with soluble or spy-VLP displayed malaria antigens. (**a**, *upper panels*) Antigen-specific IgG levels (OD Elisa) in serum from mice (n = 5 per group) immunized with a Pfs25 2xSpyTag-VLP vaccine (*filled circles*) or with a control vaccine consisting of soluble Pfs25 mixed with untagged AP205 VLPs (*open squares*). Both vaccines were formulated using aluminum hydroxide adjuvant (Statens Serum Institut, Copenhagen, Denmark). Mice were immunized on days 0, 21 and 42 and serum was collected on the indicated days after first immunization. Differences in median endpoint titers between vaccination groups were analyzed using Mann–Whitney Rank Sum test; day 14 (P < 0.01), day 35 (P < 0.01), day 56 (P < 0.01) and day 212 (P = 0.03). (**a**, *lower panels*) Similar results for the VAR2CSA based vaccines (VAR2CSA SpyCactcher-VLP and soluble VAR2CSA plus untagged AP205 VLP), which were formulated without extrinsic adjuvant. Statistical analysis; day 14 (P = 0.03), day 35 (P = 0.09), day 56 (P = 0.09) and day 137 (P = 0.03). **b** Antibody avidity was assessed on days 35 and 56 in serum samples from mice vaccinated with the Pfs25 or VAR2CSA vaccines. Avidity index values were determined by measuring the resistance of antibody-antigen complexes to 8 M urea. The avidity index was calculated as the ratio of the mean ELISA OD_490_ value of urea-treated wells to PBS control wells multiplied by 100. Mann–Whitney Rank Sum test was used for statistical comparisons. **c** The distribution of IgG1, IgG2a and IgG2b relative to the total vaccine-induced IgG response in mice (n = 5 per group) following Pfs25 or VAR2CSA immunization. Anti-Pfs25 and anti-VAR2CSA sera (left) were obtained on days 98 and 88, respectively. Mann–Whitney Rank Sum test was used for statistical comparisons

The antigen display capacities for Pfs25 and VAR2CSA were 109 and 61 proteins per VLP, respectively (Table 1; Additional file 3: Figure S2C, K). The antigen-specific IgG titer was measured by enzyme-linked immune-sorbent assay (ELISA) 2 weeks after each immunization (on days 14, 35 and 56) as well as at day 212 (Pfs25) and 137 (VAR2CSA) (Fig. 2a). The Pfs25 spy-VLP vaccine induced higher antigen-specific IgG titers than the control vaccine at all the tested time-points (P < 0.01 (day 14, 35 and 56); P = 0.03 (day 212), Mann–Whitney Rank Sum Test) (Fig. 2a). At day 212, there was a 37-fold increase in the geometric mean titer (GMT) of IgG in sera from spy-VLP vaccinated mice compared to mice vaccinated with the same amount of soluble Pfs25 plus untagged AP205 VLPs.

The GMT of VAR2CSA-specific IgG was consistently higher in the group immunized with the VAR2CSA spy-VLP vaccine compared to the control group vaccinated with uncoupled VAR2CSA. However, the difference in GMTs between the two groups was not as profound as seen in the Pfs25 study and only reached statistical significance at days 14 (P = 0.03, Mann–Whitney Rank Sum Test) and 137 (P = 0.03, Mann–Whitney Rank Sum Test) (Fig. 2a).

#### Avidity of spy-VLP vaccine induced IgG

In order to further examine qualitative differences in humoral responses, we investigated the avidity of IgG antibodies induced after immunizations with spy-VLP vaccines compared to control vaccines (uncoupled antigen + untagged AP205 VLPs). The avidity index values of serum IgG were determined by measuring the resistance of antibody-antigen complexes to 8 M urea by ELISA, as described [29]. Avidity index values can be divided into three categories denoting: high avidity (avidity index values higher than 50 %), intermediate avidity (between 30 and 50 %) and low avidity (>30 %) [29]. Prior to measuring avidity, pre-determined IgG antibody levels in individual mouse serum samples were equalized by dilution. Both the Pfs25 and VAR2CSA spy-VLP vaccines induced antigen-specific IgG with significantly higher avidity-index values compared to corresponding control vaccines P = 0.015 (Pfs25 day 35) and P = 0.032 (Pfs25 day 56) and P = 0.032 (VAR2CSA day 35) and P = 0.056 (VAR2CSA day 56), Mann–Whitney Rank Sum Test) Fig. 2b. The mean avidity index value of anti-Pfs25 sera obtained at day 35 was 46 % for the spy-VLP group, thus falling within the “intermediate avidity” category, whereas the mean value of the control group was only 22 % (i.e. low avidity). The corresponding values for day 56 were 57 % (i.e. high avidity) and 30 % (i.e. intermediate avidity) for the spy-VLP and control group, respectively. The mean avidity of antibodies induced by the VAR2CSA spy-VLP vaccine was 63 and 62 % (both high avidity) in sera obtained at day 35 and 56, respectively. In comparison, mean avidity of antibodies induced by the control vaccine was 39 and 42 % (both intermediate avidity) in sera obtained at day 35 and 56, respectively.

#### Subclass profiling of vaccine induced IgG

The relative proportion of IgG subclasses elicited in the spy-VLP vaccinated groups and the control groups was also compared. Measurements were performed on sera obtained at day 98 (Pfs25) or 88 (VAR2CSA) and calculations were based on ELISA measurements using subclass-specific (IgG1, IgG2a and IgG2b) secondary anti-mouse IgG antibodies for quantification. Anti-mouse total IgG secondary antibody was used for normalization. IgG1 was the dominant IgG subclass in all sera. Sera from Pfs25 vaccinated mice contained IgG1 and IgG2b and the spy-VLP vaccine induced significantly higher IgG2b (P = 0.03) and significantly lower (P = 0.03) IgG1 levels compared to the control vaccine (Fig. 2c, left). Sera from VAR2CSA vaccinated mice contained IgG of all subclasses. The distribution of IgG subclasses was similar in mice vaccinated with the two VAR2CSA vaccines. (Fig. 2c, right).

#### Functional activity of spy-VLP induced humoral responses

**Fig. 3:**
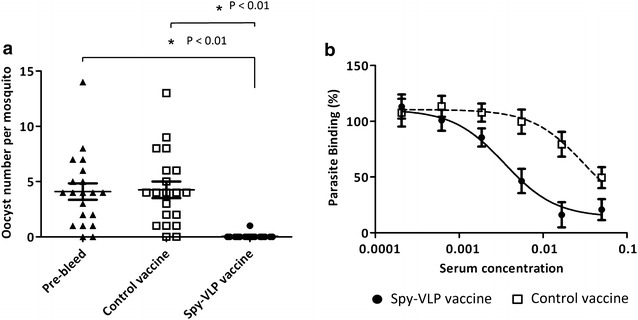
Functional activity of spy-VLP vaccine-induced humoral responses. **a** Transmission reducing activity (TRA) of anti-Pfs25 (day 56) sera following immunization of mice with the Pfs25 spy-VLP vaccine or with the control vaccine (soluble Pfs25 + untagged AP205 VLP) as described in Fig. 2a. *Y*-axis shows the number of oocysts identified in the midgut of each of 20 *A. stephensi* mosquitoes. Pre-immunization mouse serum was used as additional negative control. Mann–Whitney rank sum test was used for statistical comparisons. **b** Binding between VAR2CSA expressing infected erythrocytes and CSA in the presence of different concentrations of serum from immunized mice. Binding in the presence of serum from non-immunized mice was set to 100 %. Serum pools were from groups of 5 mice immunized with the VAR2CSA spy-VLP vaccine (*black circle*) or with control vaccine (*empty square*). The EC50 value for the spy-VLP vaccinated mice was 8.8 fold higher [3.213–14.41] than the value from mice receiving the control vaccine

For most vaccines only a fraction of the induced IgG is biologically active in inhibiting the development of the targeted microorganism and the level and overall avidity of vaccine induced IgG responses does not necessarily reflect the anti-microbial functional activity. The goal of the Pfs-25 vaccine is to block parasite development inside the mosquito. We therefore used the standard membrane feeding assay (SMFA) to measure transmission-blocking (TB) activity of the antibodies induced by the two vaccines [30]. The Pfs25 spy-VLP vaccine showed more than 99 % transmission-reducing activity (TRA) (one oocyst found in the 20 investigated mosquitos) compared to pre-immune serum (82 oocysts detected) or serum from mice immunized with the control vaccine (85 oocysts detected) (Fig. 3a). In a second study, BALB/c mice (n = 7) were immunized with the Pfs25 spy-VLP vaccine at days 0 and 14, and different concentrations of purified IgG from pooled anti-Pfs25 serum samples were subsequently tested in the SMFA assay. At the highest IgG concentration (750 µg/ml) serum from the Pfs25 spy-VLP vaccinated mice completely blocked oocyst formation (Table 2).

**Table 2:** Transmission-reducing activity of Pfs25 spy-VLP vaccine induced IgG

	Control serum	Serum from Pfs25 spy-VLP immunized mice			
Tested concentrations (µg/ml) of purified total serum IgG	750	750	250	83.3	27.8
Detected oocysts per 20 tested mosquitoes	133	0	1	5	97
Median (25 and 75 percentiles)	5.5 (3:10)	0 (0:0)	0 (0:0)	0 (0:0)	4.0 (2:6.75)
Transmission-reducing activity (%)^a^	–	100	99.2	96.2	27.1
P value^b^	–	<0.001	<0.001	<0.001	0.704

^a^Total oocyst count in mosquitoes fed with anti-Pfs25 IgG/total oocyst count in mosquitoes fed with control serum IgG × 100

^b^Mann–Whitney Rank Sum Test

Serum concentrations of 250 and 83.3 µg/ml showed more than 95 % transmission-reducing activity compared to IgG purified from ovalbumin immunized mice (Table 2).

The aim of VAR2CSA vaccines is to induce IgG, which inhibit the binding between parasite-infected erythrocytes and placental chondroitin sulfate. We therefore compared the vaccines ability to elicit IgG inhibiting the binding between VAR2CSA expressed on infected erythrocytes and chondroitin sulfate in an in vitro assay (Fig. 3b). The mean EC50 calculated from the dose–response curve of serum from VAR2CSA spy-VLP immunized mice was eightfold higher, [CI 95 % 3.213–14.41], than the mean EC50 in serum from mice vaccinated with VAR2CSA that was not bound to VLPs (Fig. 3b).

### Breaking self-tolerance by spy-VLP display

**Fig. 4:**
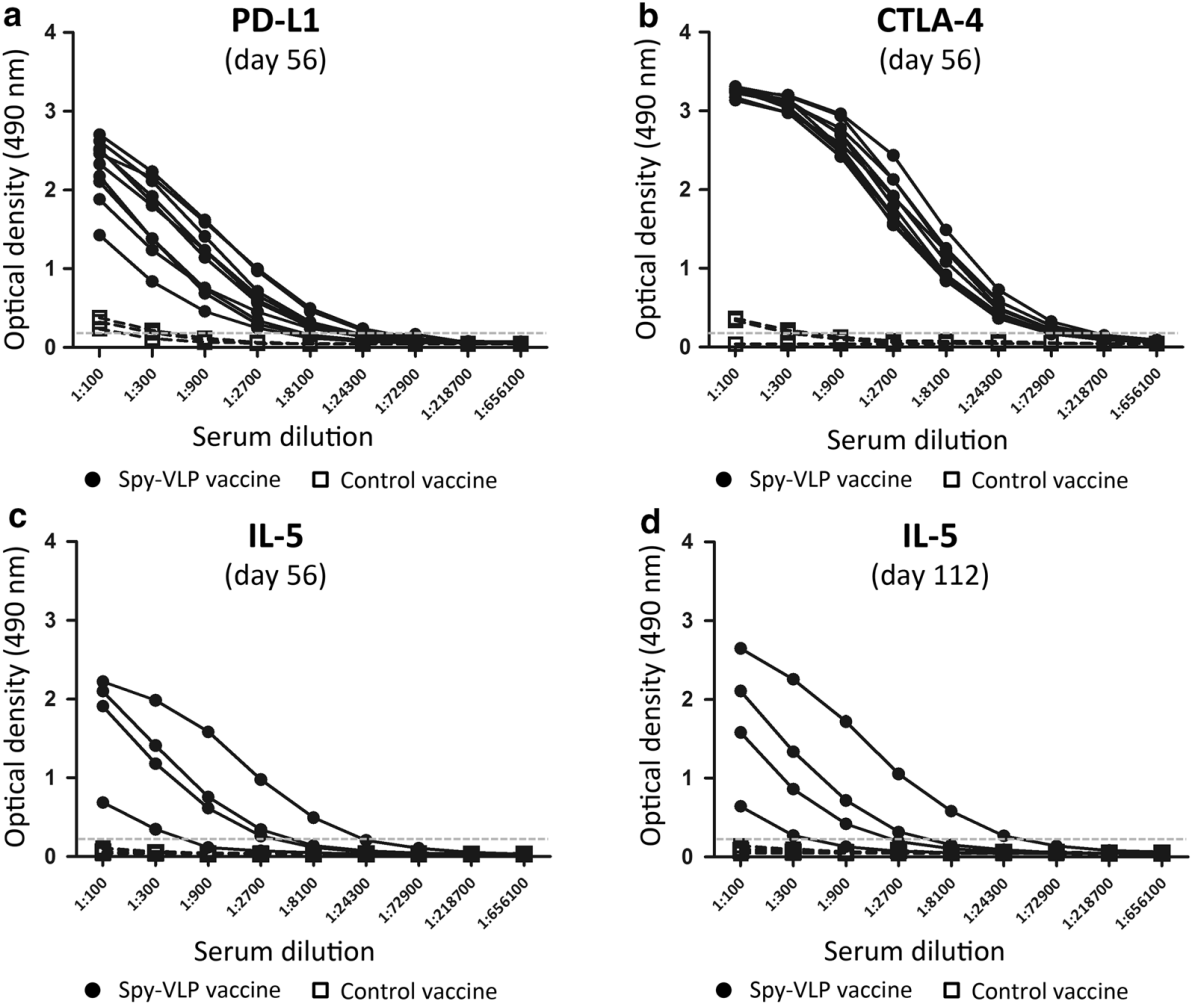
Breakage of self-tolerance. IgG autoantibody responses measured by standard ELISA. **a**, **b** C57BL/6 mice (n = 10 per group) were immunized with a PD-L1 (**a**) or CTLA-4 (**b**) SpyCatcher-VLP vaccine (*filled circles*) or with a control vaccine (n = 3 per group) consisting of similar amounts of spy-antigen mixed with untagged AP205 VLPs (*open squares*). Both vaccines were formulated using aluminum hydroxide adjuvant (Statens Serum Institut, Copenhagen, Denmark). Mice were immunized with a dose of 5 µg antigen on days 0, 21 and 42 and serum was collected on day 56 after first immunization. Median endpoint titers were compared for the PD-L1 vaccination groups (P = 0.01) and the CTLA-4 vaccination groups (P = 0.01) using Mann–Whitney Rank Sum test. **c**, **d** BALB/c mice (n = 4) were immunized with an IL-5 SpyTag-VLP vaccine or a control vaccine (soluble IL-5 + untagged AP205 VLP) (n = 5) which were both formulated with aluminum hydroxide (Statens Serum Institut, Copenhagen, Denmark). Mice were immunized on days 0, 21 and 42 with antigen doses of 5, 2.5 and 2.5 µg, respectively, and serum was collected on days 56 (**c**) and 112 (**d**). Median endpoint titers for the two vaccination groups were compared using Mann–Whitney Rank Sum test; day 56 (P = 0.2) and day 122 (P = 0.2)

To examine the capacity of the spy-VLP system to overcome B cell self-tolerance and induce autoantibody responses upon vaccination with self-antigens, mouse proteins PD-L1, CTLA-4 and interleukin-5 were recombinantly expressed with SpyTag or SpyCatcher and formulated as spy-VLP vaccines. PD-L1 and CTLA-4 down regulate T cell function. Expression of these proteins in tumors has been linked to poor prognosis and antibody-targeting of the proteins is showing promise in cancer treatment [31]. Antibody measurements of anti-sera obtained at day 56 showed that the PD-L1 and CTLA-4 spy-VLP vaccines induced significant antibody responses against the self-antigens compared to the soluble protein (P = 0.01 and P < 0.01, respectively) (Fig. 4a, b).

The therapeutic use in severe asthma of antibodies targeting IL-5 is supported by abundant data from in vitro experiments, animal models and clinical trials [32]. The SpyCatcher was fused at the N-terminus of mouse IL-5. Antibody measurements of anti-IL-5 in sera obtained at day 56 showed that the spy-VLP vaccine induced significant antibody responses against this self-antigen compared to the control vaccine (P = 0.02) (Fig. 4c). The autoantibody response elicited by the spy-VLP vaccine did not decay rapidly as the titers measured at day 112 (Fig. 4d) were similar to those measured at day 56 (P = 0.02) (Fig. 4c).

Mixing SpyCatcher-IL-5 with 2xSpyTag-VLPs resulted in a higher antigen coupling capacity than when mixing the same antigen with SpyTag-VLPs (Table 1; Additional file 3: Figure S2D). These two mixtures were administered to mice to compare IgG levels elicited by the two vaccines. The GMT titer of sera obtained at day 35 from mice vaccinated with the IL-5 2xSpyTag-VLP vaccine (GMT = 580) was 13 fold higher than for the anti-IL-5 SpyTag-VLP sera (GMT = 41) (P = 0.06, Mann–Whitney Rank Sum test), suggesting that the increased antigen coupling capacity of the 2xSpyTag VLP had an effect on the immunogenicity of the vaccine (Additional file 5: Figure S4).

## Discussion

Virus-like particles (VLPs) are effective at establishing both prophylactic and therapeutic immunity against their source virus or foreign antigens displayed on their surface. Aiming at developing a versatile VLP-based antigen display platform, we designed a panel of genetically modified spy-AP205 coat proteins, which assembled into stable, non-aggregated VLPs presenting reactive SpyTag or SpyCatcher on their surfaces. The *Acinetobacter* phage AP205 was chosen as platform as AP205 VLPs have been shown to tolerate fusion of heterologous peptides at both the N- and C-terminus of the structural protein [17] and because AP205 VLPs can be produced at low cost in *E. coli*. The spy-VLPs were assessed in terms of their applicability to bind and display antigens using the SpyCatcher/SpyTag conjugation system [16]. This protein-coupling tool offers several key advantages. SpyTag and SpyCatcher polypeptides react and form an irreversible isopeptide bond at high yield under a variety of conditions (including variation in pH, temperature or buffer composition) [16], which offer ample possibilities for antigen-specific optimization. We have previously experienced that antigen-VLP conjugates tend to form aggregates and precipitate upon mixing of antigen and VLP. Subsequent modification of buffer conditions can prevent this while still allowing efficient conjugation (data not shown). Notably, a similar split-intein conjugation system (Snooptag/SnoopCatcher) was recently reported, which may be used in a similar strategy for conjugation of antigen to pre-assembled VLPs [33].

Having developed both SpyTag and SpyCatcher fused VLPs; either binding partner can be chosen as fusion-tag to the antigen. In most cases the SpyTag peptide will be the preferred choice since the minor addition of 13 amino acid residues to the antigen is unlikely to negatively influence protein expression or polypeptide folding, which in turn could have impact on the functional capacity of elicited IgG. Interestingly, for several proteins, we observed increased expression yields after fusing the SpyCatcher to the antigen, creating an additional rational for employing SpyTag-VLPs for conjugation of SpyCatcher-fused antigens. Specifically, we were able to express soluble and correctly folded Pfs25-SpyCatcher using *E. coli* SHuffle^®^ (NEB Biolabs) strains. Notably, *E. coli* expression of soluble Pfs25, in functional conformation, has not previously been reported [34] and expression of naked Pfs25 using similar *E. coli* SHuffle^®^ cells resulted, in our hands, in formation of inclusion bodies (data not shown).

Epitope density is an important factor for B cell activation [35]. It was thus interesting to examine whether the spy-VLP system presented vaccine antigens at high density. The VLPs formed by structural proteins to which one SpyTag or SpyCatcher were fused provided 180 binding sites per VLP. We tested 11 spy-antigens in different combinations with these spy-VLP platforms. The antigen density per particle varied according to antigen and the VLP employed, but the mean display capacity was 118 antigen molecules per spy-VLP, corresponding to that antigens had been coupled to 65 % of the available VLP subunits. We had expected antigen-specific characteristics (*e.g.* net surface charge) would have a greater effect on the coupling efficiency than what was observed, but the observed correlation between antigen size and the density of antigen display on the VLPs, suggests that steric hindrance plays a role. The VLPs with 180 available antigen binding sites elicited robust IgG responses, however we also designed VLPs where each subunit protein had two SpyTags attached. These VLPs had 360 binding sites available. The immunogenicity of IL-5 was higher when administrered with the high density VLP (193 antigens coupled per VLP) than with the lower density VLP (138 antigens coupled per VLP), but the difference only achieved borderline statistical significance (P = 0.06). Due to close proximity of the SpyTags on the VLPs with 360 binding sites it is likely that smaller proteins are better able to bind effectively to these particles, whereas larger proteins face problems of steric hindrance. Thus each of the three developed spy-VLP platforms contributes to the versatility of the antigen display system.

The long-term success of a Pfs25-based transmission-blocking vaccine depends upon induction of high and sustainable levels of functional antibody that effectively block parasite development in the mosquito [36, 37]. In this regard, a single immunization with bivalent HPV16/18 L1 VLP vaccine (Cervarix) has been shown to induce protective antibody levels remaining stable for more than 48 months [38]. This has been attributed to the ordered, repetitive, and dense display of epitopes on the VLP surface. We found, that the immunogenicity of Pfs25 on spy-VLPs was high and that the anti-Pfs25 IgG levels was considerably higher 6 months post immunization compared to mice vaccinated with uncoupled protein, although IgG levels showed a tendency to decline throughout the study period. Further long-term studies are thus needed to establish if anti-Pfs25 levels will eventually plateau and, in that event, assay the transmission-blocking activity of the remaining antigen-specific IgG.

Anti-VAR2CSA IgG titers were not boosted to the same extent by the spy-VLP display as seen in the Pfs25 study. Likely, this is explained by the fact that soluble VAR2CSA is a superior immunogen i.e. the soluble protein induced relatively high antigen specific IgG titers. However, the comparably lower antigen display capacity of the VAR2CSA spy-VLP vaccine (61 antigens per VLP) may also have limited immunogenicity. Importantly, a vaccine to protect against placental malaria should be administered prior to conception i.e. to pre-puberty girls. Therefore, several years are likely to lapse between vaccination and exposure. It is thus important to note that anti-VAR2CSA IgG titers were significantly higher in the spy-VLP immunized group compared to the control when examining serum samples obtained at day 137.

VLP display had a significant positive effect on the avidity of the humoral response against both Pfs25 and VAR2CSA. The avidity index values determined for anti-Pfs25 IgG in sera from mice immunized with soluble Pfs25 formulated in aluminum hydroxide were categorized as “low” to “intermediate” affinity whereas IgG in corresponding serum samples from Pfs25 spy-VLP immunized mice were categorized as “high affinity”. The avidity of anti-VAR2CSA IgG was also higher after vaccination with VLPs compared to vaccination with uncoupled protein. This might be of importance since the avidity of anti-VAR2CSA IgG has been associated with protection against placental malaria [39]. This might seem somewhat surprising as one might predict that VLP display could lower the average avidity of induced antibodies since the multivalent interaction of the displayed antigen with B cell receptors would lead to activation of lower affinity B cells. However, the ability of virus-like display to break B cell peripheral self-tolerance and activate anergic B cells, perhaps via signaling through IgD, may contribute to the observed higher avidity. These activities would permit the generation of antibody secreting plasmablasts through tolerized naïve B cells and self-reactive intermediates generated during somatic hypermutation and this would not be the case with monomeric antigen. This situation could result in a more diverse antibody repertoire that would drive selection of higher affinity clones [40–42].

Comparison of IgG subclasses revealed a significantly (2–2.5 fold) higher induction of anti-Pfs25 IgG2b in Pfs25 spy-VLP vaccinated mice compared to the control group (such a difference was not observed in the VAR2CSA study). In mice, IgG1 is associated with a T-helper cell type 2 (Th2)-like response, while a Th1 response is associated with the induction of IgG2a, IgG2b, and IgG3 antibodies [43]. IgG2b (and IgG2a) exhibit the strongest binding to Fc receptors [44] and has a higher capacity for complement fixation than IgG1 [45]. Both Pfs25 vaccines were formulated in aluminum hydroxide (Statens Serum Institut, Copenhagen, Denmark), which is a known stimulator of Th2 responses. It is interesting that the Pfs25 spy-VLP vaccine was able to induce a significantly higher IgG2b level, indicating a boosted Th1 response. The IgG subclasses can contribute to clearance of pathogens by different mechanisms. Accordingly, the induction of a broad subclass and a balanced Th1/Th2 response might be desirable for some vaccines. AP205 VLPs expressed in *E. coli* contain host cell RNA, which can activate the innate immune system via TLR 7 and 8. Besides, endotoxin levels were not measured in the VLP preparations. It is thus a possibility that bacterial RNA and/or endotoxins have affected the profile of IgG subclasses induced by the spy-VLP and unconjugated control vaccine, respectively.

For Pfs25, the spy-VLP vaccine was far more potent than the vaccine based on soluble protein in the ability to elicit biologically functional antibodies. Indeed when our results are benchmarked against other Pfs25 vaccination studies the tested Pfs25 spy-VLP vaccine shows state-of-the-art efficacy [34, 46, 47]. Similar findings were observed for VAR2CSA, the functional parasite binding-inhibition assay showed an eight fold higher parasite binding-inhibition capacity in sera from spy-VLP vaccinated mice compared to control sera. In conclusion, the results of the Pfs25 and VAR2CSA immunization studies demonstrate proof-of-concept for the spy-VLP system to increase the functional antibody responses to complex vaccine antigens.

Display of self-antigens on VLPs is an effective approach for inducing strong antibody responses against self-antigens providing opportunities for development of therapeutic VLP-based vaccines [48–50]. We thus examined if the spy-VLP system was efficient in breaking self-tolerance. These studies, based on both cancer (PD-L1 and CTLA-4) and allergy (IL-5) associated self-antigen targets, demonstrated a consistent ability of the spy-VLP mediated antigen-display to facilitate induction of potent autoantibody responses. Previously, VLP-display induced autoantibody responses have been reported to wane over time with a typical half-life in humans and non-human primates of ~3 months [51–53] and there has been no evidence for boosting by endogenous self-antigen, probably since foreign T cell helper responses are required to stimulate antibody production. It is thus interesting that our IL-5 spy-VLP vaccine induced antigen-specific autoantibody titers, which did not show a significant decline over a period of 4 months post immunization.

Reporting work, done in parallel and unknown to us, Brune et al. recently published on the development of a SpyCatcher-AP205 VLP, which was used in a similar strategy to ours to develop VLP-vaccines based on two malaria antigens (Pfs25 and CIDR) [54]. In that study, proof-of-principle was established alone by showing increased antigen-specific IgG titers in sera obtained approximately 2 weeks after immunization of mice with the spy-VLP vaccines compared to a control vaccine. Our study extends these results by presenting different spy-VLP platforms, which are each used to characterize the systems versatility for antigen display. Furthermore, spy-VLP-induced responses in mice were analyzed over several months and included measurement of antibody titer, avidity and subclass distribution. Finally, we demonstrate proof-of-concept for the spy-VLP system to increase functional antibody responses against different pathogen antigens as wells as to overcome B cell self-tolerance.

The requirement of simple subunit-based vaccines to be administered with an extrinsic adjuvant, of which only a few have been approved for human use [55], is an impediment for vaccine development. VLP-display of vaccine antigens might mitigate this requirement and enable development of adjuvant-free vaccines. A generic VLP-display platform may moreover be instrumental for screening of vaccine candidate antigens since especially low-immunogenic or cryptic antigens (*e.g.* Pfs25, HPV L2, PfRH5) depend greatly on increased immunogenicity for their protective potential. The ability of the spy-VLP system to display whole antigens enables induction of polyclonal antibody responses, which may have a superior neutralizing capacity compared to the essentially monoclonal responses induced by epitope-based vaccines. Finally, the simple production of VLP vaccines that have been made possible by the versatile spy-VLP system raises the possibility that the spy-VLPs could be pre-manufactured and distributed as a generic tool for use in laboratories working on vaccine development against a wide variety of target antigens.

## Methods

### Design, expression and purification of spy-AP205 virus-like particles

The *Acinetobacter phage* AP205 coat protein (Gene ID: 956335) was used to design three “spy-VLPs”, presenting either SpyTag or SpyCatcher on their surface. SpyTag-VLP was constructed by adding the SpyTag peptide sequence (AHIVMVDAYKPTK) to N-terminus of the AP205 coat protein. 2xSpyTag-VLP had the SpyTag added to both the N- and C-terminus of the AP205 coat protein. SpyCatcher-VLP had the SpyCatcher protein added to the N-terminus of AP205 coat protein. The constructs were all designed to contain a flexible linker between the binding tag and the AP205 coat protein; GSGTAGGGSGS (SpyTag-VLP), GGSGS (SpyCatcher-VLP), GSGTAGGGSGS (N-terminus of 2xSpyTag-VLP) and GTASGGSGGSG (C-terminus of 2xSpyTag-VLP). Gene sequences were further modified to contain *Nco*I and *Not*I restriction sites at the N- and C-termini, respectively, and were codon-optimized for recombinant expression in *E. coli* before being synthesized by (GeneArt^®^ Life Technologies, Germany). All spy-VLP expression sequences were cloned into a pET-15b vector and transformed into One Shot^®^ BL21 Star™ (DE3) (Thermo Scientific) cells. Transformed colonies were screened by small-scale (10 mL) protein expression followed by SDS-PAGE analysis to identify the transformed clones with the highest level of protein expression. Expression was done in 3L shake flasks containing 400 mL 2xYT media (100 µg/mL ampicillin). Bacterial cultures were incubated for approximately 3 h (OD_600_ = 0.6) at 37 °C before they were induced with 1 mM IPTG and then allowed to incubated for additional 16 h at 20 °C. Cultures were harvested by centrifugation (10,000*g*) and pellets were resuspended in 1xPBS (pH = 7.2) and lysed by sonication at 80 % power with 5 pulsations for 2 × 5 min on ice. Cleared bacterial lysates were then purified by ultracentrifugation (UC) through an Optiprep™ (Sigma) step (23, 29 and 35 %) gradient, modified from [56]. In brief, 1.2 ml bacterial lysate was loaded on top of the gradient in Polyallomer Centrifuge open top tubes (11 × 60 mm) (Bechmann Coulter) and spun at 307.900 RCF (SW60Ti rotor, Beckmann Coulter) for 3.30 h at 16 °C. UC fractions were subsequently analyses by SDS-PAGE and VLP-containing fractions were pooled and dialyzed in PBS (0.02 % PS80, pH 7.2) using a 300.000 MWCO membrane (Spectrum). Dialyzed VLP samples were finally spun down at 16,000*g* for 2 min at 4 °C to remove aggregates. Protein concentration was determined by bicinchoninic acid assay (Sigma), following manufacturer’s instructions.

### Electron microscopy

Using the droplet method, an aliquot of VLPs was diluted to 0.2 mg/mL in PBS. Diluted VLPs were adsorbed to carbon and negatively stained with 2 % phosphotungstic acid (pH = 7.0) for 1 min. A grid was placed on the carbon floating on top of the 2 % phosphotungstic acid stain droplet. Excess stain was removed with filter paper. The sample was examined with a CM 100 BioTWIN electron microscope (Phillips) at an accelerating voltage of 80 kV. Photographic records were obtained on an Olympus Veleta camera.

### Particle size measurement by dynamic light scattering (DLS)

VLPs were diluted to 0.2 mg/mL in PBS and spun at 15,000*g* for 10 min at 4 °C to remove aggregates. 70 µl of each VLP sample was loaded into disposable low volume cuvettes and mounted into the DLS chamber. Size distribution was obtained by DLS measurements at 25 °C using WYATT Technology, DynaPro NanoStar, equipped with a 658 nm laser. Each sample was measured twice with 20 runs. The mean size of the most predominant particles in the population was calculated together with the % polydispersity (% Pd).

### Design, expression and purification of spy-antigen

All vaccine constructs were designed with a VLP binding-tag (SpyCatcher or SpyTag) and a hexa histidine purification tag at opposite termini of the antigen. A flexible serine/glycine linker was inserted between the spy binding-tag and the antigen. Gene sequences codon-optimized for expression in *E. coli* were modified to contain *Nco*I and *Not*I restriction sites at the N- and C-terminus, respectively. Gene sequences codon-optimized for expression in *Spodoptera frugiperda* insect cells were designed with *Bam*HI and *Not*I restriction sites at the N- and C-terminus, respectively. All sequences were synthesized by (GeneArt^®^ Life Technologies, Germany). A complete list of antigens used in this study is shown in (Additional file 2: Table S1). For expression in *E. coli* One Shot^®^ BL21 Star™ (DE3) (Thermo Scientific) or SHuffle^®^ T7 Express (C3029H New England BioLabs) cells, gene sequences were cloned in into a pET-15b vector and expressed, as described previously (see Expression and purification of spy-AP205 virus-like particles). For expression in baculovirus-transfected insect cells gene fragments were cloned into the BamHI/NotI sites of the pAcGP67A vector (BD Biosciences). To generate recombinant virus particles, linearized BakPak viral DNA (BD Biosciences) was co-transfected with pAcGP67A/Avi-L1 into Sf9 insect cells using Lipofectamine 2000 10 Reagent (Invitrogen, 11668–019) and incubated at 28 °C for 3–5 days. Recombinant baculovirus was harvested from the supernatant and used to generate a high-titer virus stock, which was used for infection of High-Five insect cells. Infected High-Five cells were incubated for 48 h at 28 °C with shaking. Expression in S2 insect cells were done as previously described [57].

### Conjugation of vaccine antigens to VLPs

To attach vaccine antigen to VLPs, spy-VLPs and spy-antigen were incubated overnight at 4 °C at a 1:1.5 M ratio. Antigen and VLPs were mixed in a standard phosphate-buffered saline buffer supplemented with 0.2 % Polysorbate 80, pH 7.2. To prevent aggregation/precipitation of the antigen-VLP conjugate it was sometimes necessary to optimize buffer conditions by adjusting the pH and/or ionic strength of the buffer (PD-L1 VLP vaccine was mixed in PBS, 0.8 M NaCl, pH 6.2). Coupling efficiencies reported in Table 1 were estimated by densitometric analysis of SDS-PAGE gels (Additional file 3: Figure S2) using ImageQuant TL software. The percentage of VLP subunits, which had been conjugated to an antigen via interaction between SpyTag and SpyCatcher (% coupling efficiency), was estimated by dividing the intensity value of the VLP subunit protein band before reaction with antigen with the corresponding intensity value after reaction with antigen, multiplied with 100. This percentage was subsequently multiplied by 180 or 360 to calculate the antigen-display capacity (number of antigens/VLP).

### Immunization of mice

Mice were immunized with the described VLP vaccines or control vaccines prepared in a similar way as the VLP vaccines, but where the Spy-tagged vaccine antigens were incubated with untagged AP205 VLPs. Thus, the only difference between the VLP vaccines and the control vaccines was that vaccine antigens were coupled to the VLPs in the VLP vaccines, whereas the control vaccines contained soluble vaccine antigen and uncoupled VLPs. Stock solutions of spy-VLPs contained 1 mg/ml spy-AP205 protein and were estimated by SDS-PAGE to be >95 % pure. All vaccines, except the VAR2CSA based vaccines, were formulated with 2 % Alhydrogel (Statens Serum Institut, Copenhagen, Denmark) to a final concentration of 2 mg/ml aluminum hydroxide. The Alhydrogel was added 1 h prior to intramuscular immunization (50 µL/mice). The VAR2CSA vaccines were diluted with PBS and administrated without adjuvant. The indicated vaccine dose refers solely to the amount of antigen. Vaccines were administered on days 0, 21 and 42. Blood samples were obtained 2 weeks after the first, second and third immunizations. Additional blood samples were collected according to the vaccine antigen as follows: Pfs25 vaccines on days 98 and 212; VAR2CSA vaccines on days 88 and 137; IL-5 vaccines on day 112. BALB/c mice were used for the VAR2CSA and IL-5 immunization studies as well as for generating anti-Pfs25 sera used in the SMFA study testing different IgG concentrations (Table 2). C57/BL6 mice were used in the CTLA-4 and PD-L1 study as well as in the other Pfs25 immunization study (Figs. 2, 3).

### Antibody response measured by standard ELISA

Serum IgG levels were measured by standard enzyme-linked immunosorbent assay (ELISA). 96-well plates (Nunc MaxiSorp) were coated over night at 4 °C with vaccine protein, without SpyTag or SpyCatcher component, (1 µg/ml in PBS). Plates were blocked with 1 % BSA buffer for 1 h at room temperature (RT). Mouse serum diluted 1:100 in blocking buffer were added in three-fold dilutions to triplicate wells and incubated for 1 h at RT. Plates were washed three times in PBS with 0.05 % TWEEN 20 in between different steps. Horseradish peroxidase (HRP)-conjugated polyclonal goat anti-mouse IgG (A16072, Life Technologies, Denmark) was diluted 1:3000 in blocking buffer and incubated for 1 h. Finally, color reactions were developed for 7 min by adding o-phenylenediamine substrate. The HRP enzymatic reaction was terminated by addition of 2.5 M H_2_SO_4_ and the optical density was measured at 490 nm using an ELISA plate reader (VersaMax Molecular Devices). Serum IgG endpoint titers were estimated using a cutoff of OD_490_ = 0.2.

### Serum IgG subclass profiling by ELISA

IgG subclass profiling of anti-Pfs25 and anti-VAR2CSA mouse sera followed a standard ELISA protocol, as described above. However, serum IgG levels were first normalized based on pre-determined ELISA OD_490_ values by dilution. Subsequent to incubation of IgG-normalized serum samples, secondary HRP-conjugated antibodies were used for detection of total mouse IgG (A16072, Life Technologies, Denmark) along with mouse IgG subclasses; IgG1 (A10551, Thermo Fisher), IgG2a (M32307, Thermo Fisher) and IgG2b (M32507, Thermo Fischer). For each serum sample, the OD_490_ value obtained by detection with subclass-specific anti-mouse IgG secondary antibodies was divided by the OD_490_ obtained by detection with anti-mouse total IgG secondary antibody. These relative measures were finally used for comparison of IgG subclass profiles in anti-Pfs25 and anti-VAR2CSA sera from spy-VLP immunized and mice vaccinated by the uncoupled soluble protein control vaccines.

### Measuring avidity of serum IgG

Recombinant Pfs25 or VAR2CSA (1 µg/ml in PBS) was coated on Nunc MaxiSorp plates overnight at 4 °C. Plates were incubated with blocking buffer (1 % BSA) for 1 h at room temperature (RT). Plates were washed three times in between different steps. Before adding the anti-sera for incubation with the capture antigen, IgG levels in individual serum samples were equalized by dilution. This was done based on pre-determined OD_490_ ELISA values. IgG-normalized serum samples were added (50 µl per well) to the ELISA plate in triplicates, and incubated for 1 h at RT. The ELISA wells were subsequently washed three times in PBS with 0.05 % TWEEN 20 and 50 µl of freshly made 8 M Urea was then added to the wells for 5 min (reference plates were incubated with PBS). ELISA plates were then washed three times with PBS (0.05 % TWEEN 20). HRP-conjugated polyclonal goat anti-mouse IgG (A16072, Life Technologies, Denmark) was diluted 1:3000 in blocking buffer and incubated for 1 h. Finally, color reactions were developed for 7 min by adding o-phenylenediamine substrate. The HRP enzymatic reaction was stopped by adding 2.5 M H_2_SO_4_ and the optical density was measured at 490 nm using an ELISA plate reader (VersaMax Molecular Devices). IgG avidity index values were calculated as the ratio of the mean OD value of urea-treated wells to PBS control wells multiplied by 100.

### Purification of IgG from anti-Pfs25 mouse serum

Total IgG was purified using Protein G columns (Pierce, USA) from anti-Pfs25 mouse serum samples. Briefly, protein G columns were equilibrated with binding buffer (Immunopure IgG, Pierce, USA) after which a 1:1 mixture of sera and binding buffer was allowed to flow through under gravity, the columns were then washed and eluted with elution buffer (Immunopure IgG, Pierce, USA). The eluted fraction was collected in 1 M Tris–HCl (pH 9.0, Teknova, USA) and transferred for buffer exchange to Amicon centrifugal filters (Millipore, USA) using PBS. The eluent was concentrated in PBS (Invitrogen, UK) and filtered using a 0.22 μm Millipore Ultrafree sterile centrifugal unit.

### Standard Membrane-Feeding Assay (SMFA)

Two independent SMFA analyses were performed. The first SMFA analysis was done to compare the transmission blocking (TB) activity of anti-Pfs25 sera obtained by immunization of mice with the Pfs25 spy-VLP vaccine or with the corresponding control vaccine. This SMFA experiment was conducted as previously described [30, 58]. Briefly, 30 μl of mouse serum obtained at day 56 was mixed with 90 μl of naïve human serum and 150 μl of in vitro gametocyte cultures of the *P. falciparum* (NF54) laboratory line. The mixture was fed to *Anopheles stephensi* mosquitoes through a membrane feeding apparatus. Pre-immune sera served as controls. Fully engorged mosquitoes were separated and held at 26 °C. Seven days later, midguts of 20 mosquitoes were examined for oocysts. The observed transmission reducing activity of serum was determined as the percentage reduction in the median oocyst number in test samples compared to that in controls. The experiment was considered valid when at least 85 % of the mosquitoes feeding on control sera were infected. The other SMFA study, testing different concentrations of IgG purified from anti-Pfs25 spy-VLP mouse sera, was done as described [59]. Briefly, mature *P. falciparum* strain NF54 Stage V gametocytes (adjusted to parasiteamia of 0.15 %  ±  0.05 %) were mixed with different concentrations of purified IgG from Pfs25 spy-VLP vaccinated mice. This mixture was then fed to 4–6 day old starved female *A. stephensi* (SDA 500) mosquitoes via a parafilm^®^ membrane. The mosquitoes were maintained at 26  °C and 80 % relative humidity. After 7 days mosquitoes (n = 20 per group) were dissected and the number of oocysts counted per mosquito midgut was recorded. Percent reduction in infection intensity was calculated relative to the respective control IgG (anti-ovalbumin mouse serum) tested in the same assay.

### Inhibition of binding assays

*Plasmodium falciparum* (FCR3 genotype) parasites were maintained in culture as described [60]. Parasites were panned on BeWo cells to select for a chondroitin sulphate A (CSA)-binding phenotype, as described [61]. Parasite DNA was labeled with Tritium by overnight incorporation of titrated hypoxanthine. A 96-well plate (Falcon) was coated with 2 μg/ml of Decorin (Sigma-Aldrich) overnight and blocked with 2 % bovine serum albumin (Sigma) as described [60]. Tritium labeled late-stage infected erythrocytes (IEs) were MACS purified and added to the 96-well plate in a concentration of 200,000 cells per well. Titrations of serum were added in a total volume of 100 μl in triplicate wells. After incubation for 90 min at 37 °C, unbound IEs were washed away by a pipetting robot (Beckman-Coulter). The remaining IEs were harvested onto a filter plate (Perkin-Elmer). After addition of scintillation fluid (Perkin-Elmer) the counts per minute (CPM) recording the number of non-inhibited IE was determined by liquid scintillation counting on a Topcount NXT (Perkin-Elmer). Data were adjusted to percentage of binding by dividing test result with the mean value of wells with IE incubated without serum.

### Statistical analysis

All statistical analysis was done using non-parametric, two tailed, Mann–Whitney Rank Sum Test. Statistical significance was defined as P < 0.05.

## Ethical statements

The animal studies were approved by the Danish Animal Experiments Inspectorate. Approval number: 2013-15-2934-00902/BES.

## Additional files

10.1186/s12951-016-0181-1 Recombinant expression of spy-VLP in *E. coli*. Reduced SDS-PAGE showing unmodified AP205 structural protein and structural AP205 proteins modified by Spy elements. Protein bands match the theoretical sizes of SpyCatcher-AP205 (27 kDa), 2xSpyTag-AP205 (18.5 kDa), SpyTag-AP205 (16.5 kDa) and unmodified AP205 (14.5 kDa).
10.1186/s12951-016-0181-1 Tested spy-vaccine antigens.
10.1186/s12951-016-0181-1 Estimated coupling efficiencies of spy-VLP/antigen reactions. SDS-PAGE gels demonstrating that SpyTag or SpyCatcher fused antigens had reacted with corresponding spy-VLPs. (A) Survivin, (B) CIDR, (C) IL-5, (D) Pfs25, (E) Ag85A, (F) CSP, (G) HER2, (H) PCSK9, (I) CTLA-4, (J) PD-L1 and (K) VAR2CSA. Reactions were incubated for at least 12 h at 4OC.
10.1186/s12951-016-0181-1 SDS-PAGE and Western blot of Pfs25-SpyCatcher. The figure shows a SDS-PAGE of recombinant Pfs25-SpyCatcher run under reducing and non-reducing conditions (left) and a corresponding western blot (right) where the Pfs25-Spycatcher was detected using mAb 4B7 antibody followed by hrp-conjugated goat anti-mouse secondary antibody.
10.1186/s12951-016-0181-1 Comparison of the immunogenicity of IL-5 SpyTag-VLP and IL-5 2xSpyTag-VLP vaccines. BALB/c mice were immunized with 5 µg antigen (SpyCatcher-IL-5) mixed with either SpyTag-VLP with 180 binding motifs (n = 4, open squares) or 2xSpyTag-VLP (n = 5, filled circles) with 360 potential binding motifs. Anti-IL-5 IgG titers were determined by standard ELISA using sera obtained two weeks the 3rd immunization. Anti-IL-5 IgG titers were higher in mice immunized with the 2xSpyTag-VLP vaccine (filled circles) compared to the SpyTag-VLP vaccine (open squares). The difference approached statistical significance (P = 0.06, Mann–Whitney Rank Sum test).

## References

[1] Plotkin SA, Plotkin SL (2011). The development of vaccines: how the past led to the future. Nat Rev Microbiol.

[2] Chackerian B (2007). Virus-like particles: flexible platforms for vaccine development. Expert Rev Vaccines.

[3] Cubas R, Zhang S, Kwon SK, Sevick-Muraca EM, Li M, Chen CY (2009). Virus-like particle (VLP) lymphatic trafficking and immune response generation after immunization by different routes. J Immunother.

[4] Bachmann MF, Jennings GT (2010). Vaccine delivery: a matter of size, geometry, kinetics and molecular patterns. Nat Rev Immunol.

[5] Bachmann MF, Rohrer UH, Kündig TM, Bürki K, Hengartner H, Zinkernagel RM (1993). The influence of antigen organization on B cell responsiveness. Science.

[6] Hua Z, Hou B (2013). TLR signaling in B cell development and activation. Cell Mol Immunol.

[7] Schödel F, Wirtz R, Peterson D, Hughes J, Warren R, Sadoff J (1994). Immunity to malaria elicited by hybrid hepatitis B virus core particles carrying circumsporozoite protein epitopes. J Exp Med.

[8] Kratz PA, Böttcher B, Nassal M (1999). Native display of complete foreign protein domains on the surface of hepatitis B virus capsids. Proc Natl Acad Sci USA.

[9] Pumpens P, Grens E (2001). HBV core particles as a carrier for B cell/T cell epitopes. Intervirology.

[10] Smith MT, Hawes AK, Bundy BC (2013). Reengineering viruses and virus-like particles through chemical functionalization strategies. Curr Opin Biotechnol.

[11] Sapsford KE, Algar WR, Berti L, Gemmill KB, Casey BJ, Oh E (2013). Functionalizing nanoparticles with biological molecules: developing chemistries that facilitate nanotechnology. Chem Rev.

[12] Pattenden LK, Middelberg APJ, Niebert M, Lipin DI (2005). Towards the preparative and large-scale precision manufacture of virus-like particles. Trends Biotechnol.

[13] Koho T, Ihalainen TO, Stark M, Uusi-Kerttula H, Wieneke R, Rahikainen R (2015). His-tagged norovirus-like particles: a versatile platform for cellular delivery and surface display. Eur J Pharm Biopharm.

[14] Thrane S, Janitzek CM, Agerbæk MØ, Ditlev SB, Resende M, Nielsen MA (2015). A novel virus-like particle based vaccine platform displaying the placental malaria antigen VAR2CSA. PLoS One.

[15] Jegerlehner A, Tissot A, Lechner F, Sebbel P, Erdmann I, Kündig T (2002). A molecular assembly system that renders antigens of choice highly repetitive for induction of protective B cell responses. Vaccine.

[16] Zakeri B, Fierer JO, Celik E, Chittock EC, Schwarz-Linek U, Moy VT (2012). Peptide tag forming a rapid covalent bond to a protein, through engineering a bacterial adhesin. Proc Natl Acad Sci USA.

[17] Tissot AC, Renhofa R, Schmitz N, Cielens I, Meijerink E, Ose V (2010). Versatile virus-like particle carrier for epitope based vaccines. PLoS ONE.

[18] Wu Y, Narum DL, Fleury S, Jennings G, Yadava A (2015). Particle-based platforms for malaria vaccines. Vaccine..

[19] Moreno A, Joyner C (2015). ScienceDirect Malaria vaccine clinical trials : what’ s on the horizon. Curr Opin Immunol.

[20] Wu Y, Ellis RD, Shaffer D, Fontes E, Malkin EM, Mahanty S (2008). Phase 1 trial of malaria transmission blocking vaccine candidates Pfs25 and Pvs 25 formulated with montanide ISA 51. PLoS ONE..

[21] Kaslow DC (2002). Transmission-blocking vaccines. Chem Immunol.

[22] Coban C, Ishii KJ, Stowers AW, Keister DB, Klinman DM, Kumar N (2004). Effect of CpG oligodeoxynucleotides on the immunogenicity of Pfs25, a *Plasmodium falciparum* transmission-blocking vaccine antigen. Infect Immun.

[23] Stura EA, Kang AS, Stefanko RS, Calvo JC, Kaslow DC, Satterthwait AC (1994). Crystallization, sequence and preliminary crystallographic data for transmission-blocking anti-malaria Fab 4B7 with cyclic peptides from the Pfs25 protein of *P. falciparum*. Acta Crystallogr D Biol Crystallogr.

[24] Salanti A, Staalsoe T, Lavstsen T, Jensen ATR, Sowa MPK, Arnot DE (2003). Selective upregulation of a single distinctly structured var gene in chondroitin sulphate A-adhering *Plasmodium falciparum* involved in pregnancy-associated malaria. Mol Microbiol.

[25] Andersen P, Nielsen MA, Resende M, Rask TS, Dahlbäck M, Theander T (2008). Structural insight into epitopes in the pregnancy-associated malaria protein VAR2CSA. PLoS Pathog.

[26] Staalsoe T, Shulman CE, Bulmer JN, Kawuondo K, Marsh K, Hviid L (2004). Variant surface antigen-specific IgG and protection against clinical consequences of pregnancy-associated *Plasmodium falciparum* malaria. Lancet.

[27] Nielsen MA, Resende M, de Jongh WA, Ditlev SB, Mordmüller B, Houard S (2015). The influence of sub-unit composition and expression system on the functional antibody response in the development of a VAR2CSA based *Plasmodium falciparum* placental malaria vaccine. PLoS ONE.

[28] Dahlbäck M, Jørgensen LM, Nielsen MA, Clausen TM, Ditlev SB, Resende M (2011). The chondroitin sulfate A-binding site of the VAR2CSA protein involves multiple N-terminal domains. J Biol Chem.

[29] Hedman K, Seppälä I (1988). Recent rubella virus infection indicated by a low avidity of specific IgG. J Clin Immunol.

[30] Lensen A, Van Druten J, Bolmer M, Van Gemert G, Eling W, Sauerwein R (1996). Measurement by membrane feeding of reduction in Plasmodium falciparum transmission induced by endemic sera. Trans R Soc Trop Med Hyg.

[31] Ott PA, Hodi FS, Robert C (2013). CTLA-4 and PD-1/PD-L1 blockade: new immunotherapeutic modalities with durable clinical benefit in melanoma patients. Clin Cancer Res.

[32] Garcia G, Taillé C, Laveneziana P, Bourdin A, Chanez P, Humbert M (2013). Anti-interleukin-5 therapy in severe asthma. Eur Respir Rev.

[33] Kingdom U, Business M, Corporation S, Sup EN, Cedex L, Kingdom U (2016). Programmable polyproteams built using twin peptide superglues. PNAS..

[34] Kumar R, Angov E, Kumar N (2014). Potent malaria transmission-blocking antibody responses elicited by *Plasmodium falciparum* Pfs25 expressed in Escherichia coli after successful protein refolding. Infect Immun.

[35] Jegerlehner A, Storni T, Lipowsky G, Schmid M, Pumpens P, Bachmann MF (2002). Regulation of IgG antibody responses by epitope density and CD21-mediated costimulation. Eur J Immunol.

[36] Barr BPJ, Green KM, Gibson HL, Bathurst IC, Quakyi IA, Kaslow DC (1991). Recombinant Pfs25 protein of *Plasmodium falciparum* elicits malaria transmission-blocking immunity in experimental animals. J Exp Med.

[37] Miura K, Keister DB, Muratova OV, Sattabongkot J, Long CA, Saul A (2007). Transmission-blocking activity induced by malaria vaccine candidates Pfs25/Pvs25 is a direct and predictable function of antibody titer. Malar J.

[38] Safaeian M, Porras C, Pan Y, Kreimer A, Schiller JT, Gonzalez P (2013). Durable antibody responses following one dose of the bivalent human papillomavirus L1 virus-like particle vaccine in the Costa Rica vaccine trial. Cancer Prev Res.

[39] Tutterrow YL, Salanti A, Avril M, Smith JD, Pagano IS, Ako S (2012). High Avidity Antibodies to full-length VAR2CSA correlate with absence of Placental malaria. PLoS ONE.

[40] Schiller J, Chackerian B (2014). Why HIV virions have low numbers of envelope spikes: implications for vaccine development. PLoS Pathog.

[41] Chackerian B, Durfee MR, Schiller JT (2008). Virus-like display of a neo-self antigen reverses B cell anergy in a B cell receptor transgenic mouse model. J Immunol..

[42] Übelhart R, Hug E, Bach MP, Wossning T, Dühren-von Minden M, Horn AHC (2015). Responsiveness of B cells is regulated by the hinge region of IgD. Nat Immunol.

[43] Germann T, Bongartz M, Dlugonska H, Hess H, Schmitt E, Kolbe L (1995). Interleukin-12 profoundly up-regulates the synthesis of antigen-specific complement-fixing IgG2a, IgG2b and IgG3 antibody subclasses in vivo. Eur J Immunol.

[44] Ravetch JV, Kinet JP (1991). Fc receptors. Annu Rev Immunol.

[45] Neuberger MS, Rajewsky K (1981). Activation of mouse complement by monoclonal mouse antibodies. Eur J Immunol.

[46] Li Y, Leneghan DB, Miura K, Nikolaeva D, Brian IJ, Dicks MDJ (2015). Enhancing immunogenicity and transmission-blocking activity of malaria vaccines by fusing Pfs25 to IMX313 multimerization technology. Nat Publ Gr.

[47] Kubler-Kielb J, Majadly F, Wu Y, Narum DL, Guo C, Miller LH (2007). Long-lasting and transmission-blocking activity of antibodies to *Plasmodium falciparum* elicited in mice by protein conjugates of Pfs25. Proc Natl Acad Sci USA.

[48] Tissot AC, Maurer P, Nussberger J, Sabat R, Pfister T, Ignatenko S (2008). Effect of immunisation against angiotensin II with CYT006-AngQb on ambulatory blood pressure: a double-blind, randomised, placebo-controlled phase IIa study. Lancet.

[49] Ambühl PM, Tissot AC, Fulurija A, Maurer P, Nussberger J, Sabat R (2007). A vaccine for hypertension based on virus-like particles: preclinical efficacy and phase I safety and immunogenicity. J Hypertens.

[50] Fettelschoss A, Zabel F, Bachmann MF (2014). Vaccination against Alzheimer disease: an update on future strategies. Hum Vaccin Immunother.

[51] Bachmann MF, Dyer MR (2004). Therapeutic vaccination for chronic diseases: a new class of drugs in sight. Nat Rev Drug Discov..

[52] Chackerian B, Briglio L, Albert PS, Lowy DR, Schiller JT (2004). Induction of autoantibodies to CCR5 in macaques and subsequent effects upon challenge with an R5-tropic simian/human immunodeficiency virus. J Virol.

[53] Van Rompay KKA, Hunter Z, Jayashankar K, Peabody J, Montefiori D, LaBranche CC (2014). A vaccine against CCR5 protects a subset of macaques upon intravaginal challenge with simian immunodeficiency virus SIVmac251. J Virol.

[54] Brune KD, Leneghan DB, Brian IJ, Ishizuka AS, Bachmann MF, Draper SJ (2016). Plug-and-Display: decoration of Virus-like particles via isopeptide bonds for modular immunization. Sci Rep.

[55] Pashine A, Valiante NM, Ulmer JB (2005). Targeting the innate immune response with improved vaccine adjuvants. Nat Med.

[56] Buck CB, Pastrana DV, Lowy DR, Schiller JT (2004). Efficient intracellular assembly of papillomaviral vectors. J Virol.

[57] Wright KE, Hjerrild KA, Bartlett J, Douglas AD, Jin J, Brown RE (2014). Structure of malaria invasion protein RH5 with erythrocyte basigin and blocking antibodies. Nature.

[58] Ponnudurai T, Lensen AH, Van Gemert GJ, Bensink MP, Bolmer M, Meuwissen JH (1989). Infectivity of cultured *Plasmodium falciparum* gametocytes to mosquitoes. Parasitology.

[59] Miura K, Deng B, Tullo G, Diouf A, Moretz SE, Locke E (2013). Qualification of standard membrane-feeding assay with *Plasmodium**falciparum* malaria and potential improvements for future assays. PLoS ONE..

[60] Nielsen MA, Pinto VV, Resende M, Dahlbäck M, Ditlev SB, Theander TG (2009). Induction of adhesion-inhibitory antibodies against placental *Plasmodium falciparum* parasites by using single domains of VAR2CSA. Infect Immun.

[61] Haase RN, Megnekou R, Lundquist M, Ofori MF, Hviid L, Staalsoe T (2006). *Plasmodium falciparum* parasites expressing pregnancy-specific variant surface antigens adhere strongly to the choriocarcinoma cell line BeWo. Infect Immun.

