# Supplementary figures and images for: Bacterial superglue enables easy development of efficient virus-like particle based vaccines
Source: J Nanobiotechnology. 2016 Apr 27;14:30. doi: 10.1186/s12951-016-0181-1 (PMC4847360; doi:10.1186/s12951-016-0181-1)

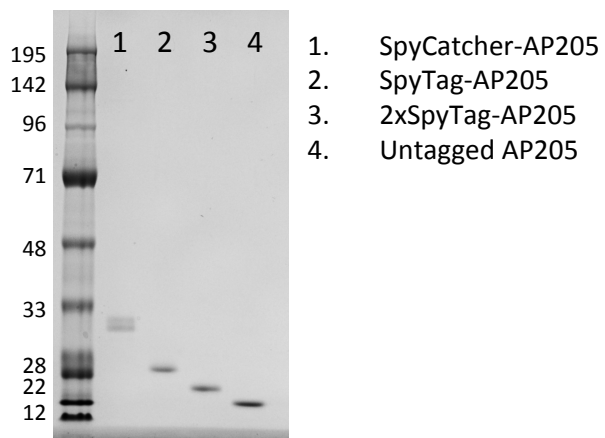

Supplement: Supplementary file 1 — 10.1186/s12951-016-0181-1 Recombinant expression of spy-VLP in E. coli. Reduced SDS-PAGE showing unmodified AP205 structural protein and structural AP205 proteins modified by Spy elements. Protein bands match the theoretical sizes of SpyCatcher-AP205 (27 kDa), 2xSpyTag-AP205 (18.5 kDa), SpyTag-AP205 (16.5 kDa) and unmodified AP205 (14.5 kDa). [file 12951_2016_181_MOESM1_ESM.pdf]

A

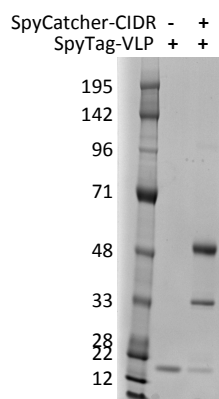

B

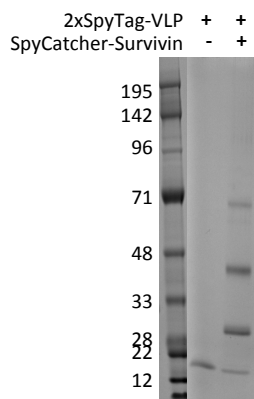

C

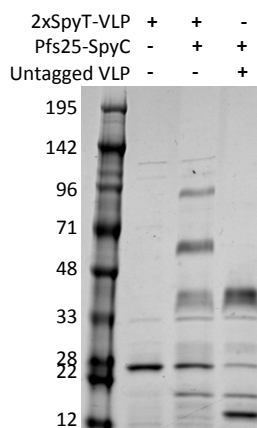

D

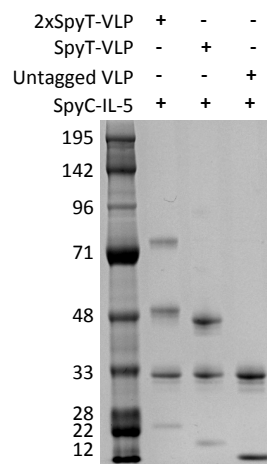

E

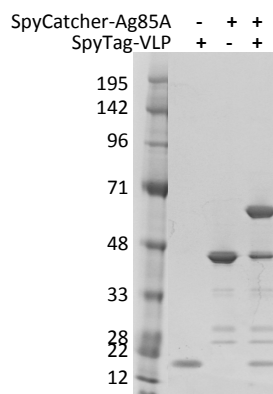

F

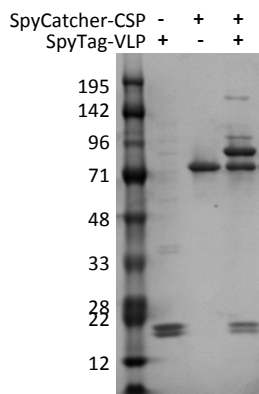

G

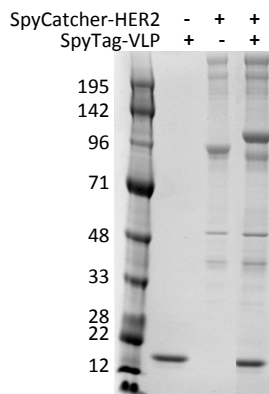

H

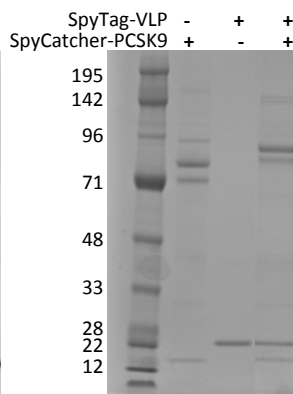

I

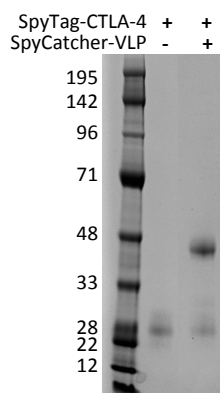

J

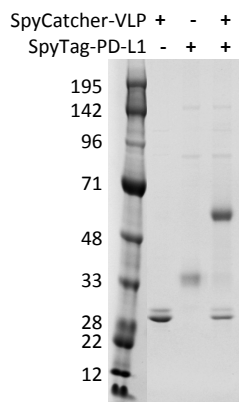

K

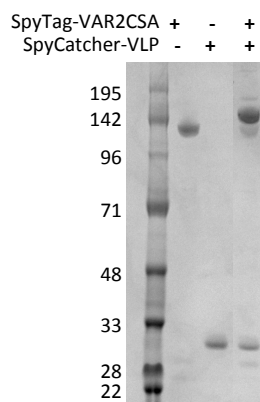

Supplement: Supplementary file 3 — 10.1186/s12951-016-0181-1 Estimated coupling efficiencies of spy-VLP/antigen reactions. SDS-PAGE gels demonstrating that SpyTag or SpyCatcher fused antigens had reacted with corresponding spy-VLPs. (A) Survivin, (B) CIDR, (C) IL-5, (D) Pfs25, (E) Ag85A, (F) CSP, (G) HER2, (H) PCSK9, (I) CTLA-4, (J) PD-L1 and (K) VAR2CSA. Reactions were incubated for at least 12 h at 4OC. [file 12951_2016_181_MOESM3_ESM.pdf]

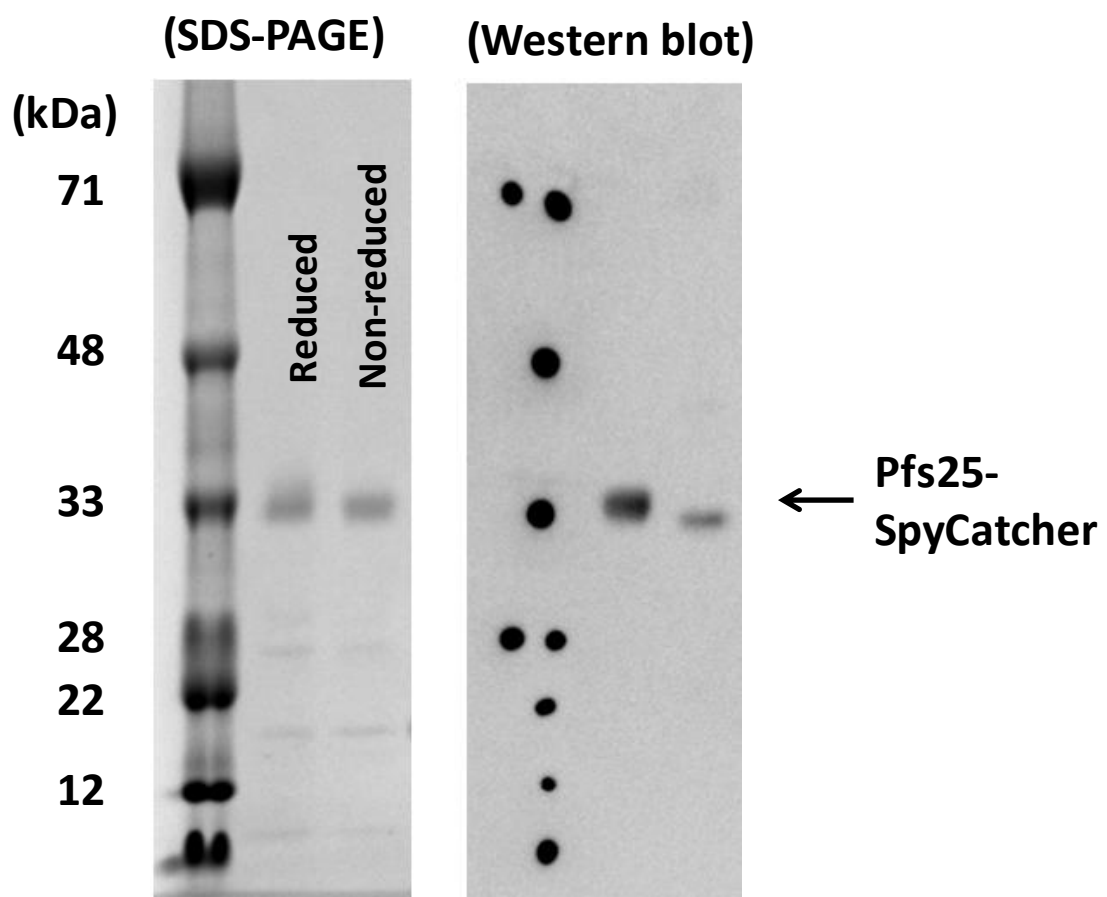

Supplement: Supplementary file 4 — 10.1186/s12951-016-0181-1 SDS-PAGE and Western blot of Pfs25-SpyCatcher. The figure shows a SDS-PAGE of recombinant Pfs25-SpyCatcher run under reducing and non-reducing conditions (left) and a corresponding western blot (right) where the Pfs25-Spycatcher was detected using mAb 4B7 antibody followed by hrp-conjugated goat anti-mouse secondary antibody. [file 12951_2016_181_MOESM4_ESM.pdf]

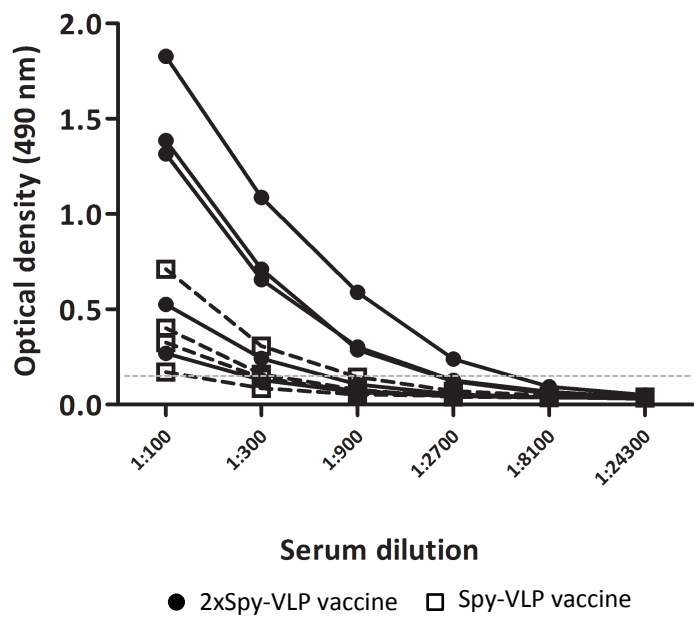

Supplement: Supplementary file 5 — 10.1186/s12951-016-0181-1 Comparison of the immunogenicity of IL-5 SpyTag-VLP and IL-5 2xSpyTag-VLP vaccines. BALB/c mice were immunized with 5 µg antigen (SpyCatcher-IL-5) mixed with either SpyTag-VLP with 180 binding motifs (n = 4, open squares) or 2xSpyTag-VLP (n = 5, filled circles) with 360 potential binding motifs. Anti-IL-5 IgG titers were determined by standard ELISA using sera obtained two weeks the 3rd immunization. Anti-IL-5 IgG titers were higher in mice immunized with the 2xSpyTag-VLP vaccine (filled circles) compared to the SpyTag-VLP vaccine (open squares). The difference approached statistical significance (P = 0.06, Mann–Whitney Rank Sum test). [file 12951_2016_181_MOESM5_ESM.pdf]
