# Supplementary material for: Bacterial superglue enables easy development of efficient virus-like particle based vaccines
Source: J Nanobiotechnology. 2016 Apr 27;14:30. doi: 10.1186/s12951-016-0181-1 (PMC4847360; doi:10.1186/s12951-016-0181-1)
Supplement: Supplementary file 2 — 10.1186/s12951-016-0181-1 Tested spy-vaccine antigens. [file 12951_2016_181_MOESM2_ESM.pdf]

Table S1. Tested Spy-Vaccine Antigens

**Spy-antigen**	**Size (kDa)**	**Expression system**	**Organism**	**Genebank Accession number**
HIS-CTLA-4-SpyTag	15	Baculovirus	*Mus musculus*	NP_033973.2
HIS-PD-L1-SpyTag	27	Baculovirus	*Mus musculus*	NP_068693.1
SpyCatcher-Survivin-HIS	30	Baculovirus	*Mus musculus*	NP_033819.1
HIS-SpyCatcher-IL-5	33	Baculovirus	*Mus musculus*	NP_034688.1
SpyCatcher-HER2-HIS	83	Baculovirus	*Homo Sapiens*	AAA75493.1
PCSK9-SpyCatcher-HIS	84	Baculovirus	*Homo Sapiens*	NP_777596.2
SpyCatcher-CIDR-HIS	32	Baculovirus	*P. falciparum*	XP_001349512.1
HIS-CSP-SpyCatcher	53	Baculovirus	*P. falciparum*	XP_001351122.1
SpyTag-VAR2CSA (domain region DBL1-ID2a)-HIS	118	E. coli SHuffle® C3029H	*P. falciparum*	ADG23053.1
HIS-Pfs25- SpyCatcher	40	E. coli SHuffle® C3029H	*P. falciparum*	XP_001347587.1
SpyCatcher-Ag85A-HIS	48	One Shot® BL21 Star™ (DE3)	*M. tuberculosis*	AAK48277.1
